# Potential Pathways for Circadian Dysfunction and Sundowning-Related Behavioral Aggression in Alzheimer’s Disease and Related Dementias

**DOI:** 10.3389/fnins.2020.00910

**Published:** 2020-09-03

**Authors:** William D. Todd

**Affiliations:** Program in Neuroscience, Department of Zoology and Physiology, University of Wyoming, Laramie, WY, United States

**Keywords:** circadian, aggression, sundowning, Alzheimer’s disease, dementia, agitation

## Abstract

Patients with Alzheimer’s disease (AD) and related dementias are commonly reported to exhibit aggressive behavior and other emotional behavioral disturbances, which create a tremendous caretaker burden. There has been an abundance of work highlighting the importance of circadian function on mood and emotional behavioral regulation, and recent evidence demonstrates that a specific hypothalamic pathway links the circadian system to neurons that modulate aggressive behavior, regulating the propensity for aggression across the day. Such shared circuitry may have important ramifications for clarifying the complex interactions underlying “sundowning syndrome,” a poorly understood (and even controversial) clinical phenomenon in AD and dementia patients that is characterized by agitation, aggression, and delirium during the late afternoon and early evening hours. The goal of this review is to highlight the potential output and input pathways of the circadian system that may underlie circadian dysfunction and behavioral aggression associated with sundowning syndrome, and to discuss possible ways these pathways might inform specific interventions for treatment. Moreover, the apparent bidirectional relationship between chronic disruptions of circadian and sleep-wake regulation and the pathology and symptoms of AD suggest that understanding the role of these circuits in such neurobehavioral pathologies could lead to better diagnostic or even preventive measures.

## Introduction

**FIGURE 1:**
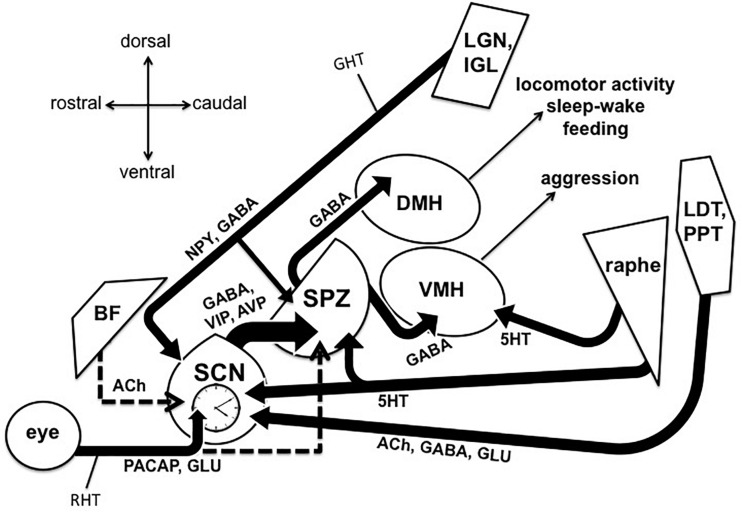
Output and input pathways of the central circadian timing system in the mammalian brain that may be involved sundowning–related behavioral aggression and circadian dysfunction in Alzheimer’s disease and related dementias. The master circadian pacemaker, is the suprachiasmatic nucleus (SCN) of the hypothalamus. The SCN releases the fast neurotransmitter GABA, as well as several peptides including vasoactive intestinal peptide (VIP) and argine vasopressin (AVP) from its major axonal output pathway to the nearby subparaventricular zone (SPZ). The GABAergic SPZ regulates rhythms of locomotor activity, sleep-wake, and feeding via pathway to the dorsomedial hypothalamus (DMH), and regulates rhythms of aggression propensity via a pathway to the ventromedial hypothalamus (VMH). The SCN is entrained to the daily light-dark cycle by input from intrinsically photosensitive retinal ganglion cells, which release pituitary adenylate cyclase activating polypeptide (PACAP) and glutamate (GLU) via the retinohypothalamic tract (RHT). The RHT also densely innervates the SPZ in most nocturnal mammals, but provides little or no innervation of the SPZ in many diurnal mammals, including humans (indicated by dashed line). A cholinergic (ACh) input to the SCN from the basal forebrain has been suggested in rats, but is absent in mice (indicated by dashed line). Cholinergic input to the SCN has also been reported from the laterodorsal tegmentum (LDT), pedunculopontine tegmentum (PPT) complex, which also releases GABA and GLU. Serotonergic (5HT) inputs to both the SCN and SPZ have been reported from the midbrain raphe complex. Finally, the geniculo-hypothalamic tract (GHT), originating from the retinoreceipient (not shown here) ventral lateral geniculate nucleus (LGN) and intergeniculate leaflet (IGL) of the thalamus, provides an input of GABA and neuropeptide Y (NPY) to both the SCN and SPZ. Structures are not drawn to scale.

Behavioral aggression and circadian dysfunction are both prevalent in several neural disorders (Todd and Machado, 2019), including Alzheimer’s disease (AD) and related dementias, and there has been an abundance of work over the last decade highlighting the general importance of circadian function on the regulation of mood and emotional behavior, including aggression (Bronsard and Bartolomei, 2013; Hood and Amir, 2018; Taylor and Hasler, 2018; Logan and McClung, 2019; Ketchesin et al., 2020). For example, circadian disruptions such as rotating shift work and jet lag due to transmeridian travel have been shown to precipitate or exacerbate mood symptoms (Asaoka et al., 2013; Kalmbach et al., 2015; Inder et al., 2016). More specifically, social jet lag (defined as a discrepancy between the body’s internal circadian clock and the actual sleep schedule) has been associated with increased physical and verbal aggression (Randler and Vollmer, 2013; Lin and Yi, 2015). Converging evidence also supports the notion that evening chronotypes exhibit a greater predisposition for behavioral aggression (Schlarb et al., 2014; Deibel et al., 2020). Recent work in transgenic mice also suggests that the master circadian pacemaker, located within the suprachiasmatic nucleus (SCN) of the anterior hypothalamus, directly modulates a rhythm in the propensity for aggressive behavior via a polysynaptic pathway contained entirely in the hypothalamus (Todd et al., 2018). Todd et al. (2018) showed that a functionally connected circuit from the SCN, through the nearby subparaventricular zone (SPZ), gates the activity of neurons within the ventromedial hypothalamus (VMH) that drive aggressive behavior (the SCN → SPZ → VMH pathway, see Figure 1). This pathway may be a substrate through which circadian dysfunction can lead to increased aggression, both acutely and chronically in disorders that are characterized by circadian disruption and high levels of aggression and agitation.

Agitation and aggression, circadian dysfunction, and several other non-cognitive symptoms of AD and dementia seem to point to an underlying disruption in the hypothalamus (Ishii and Iadecola, 2015; Hiller and Ishii, 2018), even though brainstem and cortical structures are normally the foci of most neuropathological investigations concerning these disorders. While circadian disruption of sleep-wake and other rhythms is a typical component of normal healthy aging, such dysfunction is greatly exacerbated in neurodegenerative disorders such as AD and dementia. Indeed, a growing body of evidence suggests a bidirectional interaction between the circadian system, AD pathology, and the progression of the disease (Musiek, 2015; Videnovic and Zee, 2015; Musiek and Holtzman, 2016; Duncan, 2020). Since such neurodegenerative disorders clearly disrupt the circadian rhythmicity of sleep-wake, it is likely that they also disrupt the circadian regulation of emotional processing and aggression propensity as well. Indeed, the interaction between the circadian system and processes modulating aggression may be a key contributor to the clinical phenomenon known as “sundowning syndrome”, which is commonly reported in AD and dementia patients. Sundowning is characterized by increased confusion and emotional behavioral disruptions, such as agitation and aggression, particularly during the late afternoon and early evening hours (Bachman and Rabins, 2006; Khachiyants et al., 2011; Bedrosian and Nelson, 2013; Canevelli et al., 2016). This syndrome can create a major burden on both patients and caretakers, with organizations such as the Alzheimer’s Association and the National Institute on Aging providing online caretaker resources to help them better cope with sundowning symptoms (Aging, 2017; Association, 2020). Indeed, sundowning symptoms have been cited as among the most important factors leading to the decision to seek institutionalization (Pollak and Perlick, 1991; Hope et al., 1998).

Sundowning was first described in the medical literature over 80 years ago as “senile nocturnal delirium” (Cameron, 1941), when D. Ewen Cameron noted an exacerbation of delirium and agitation that occurred within an hour of placing dementia patients into a darkened room. The term “sundowning syndrome,” due to the phenomenon’s association with the onset of daily darkness, was first coined in the late 1980s by Lois K. Evans, who described it as a recurring condition among institutionalized older adults similar to delirium, but lasting much longer (Evans, 1987). However, since that time, the relevant literature on sundowning has been relatively scarce, and the underlying pathophysiology of the syndrome remains enigmatic. Perhaps one of the primary reasons sundowning remains poorly understood is that the symptoms and criteria used to define it have differed widely across groups (Bachman and Rabins, 2006; Canevelli et al., 2016). For instance, some groups have focused more on the emotional components of the syndrome, some more on the increased nocturnal locomotor activity such as wandering, whereas fewer have described sundowning as primarily a sleep-related disturbance (Boronat et al., 2019). It is also important to note that sundowning is not an official diagnosis (it does not appear in the DSM-5), but rather a loose grouping of symptoms. These challenges probably contribute to the wide range of prevalence reported for sundowning across studies, with some studies reporting as high as 60%, while others reporting as low as 2.5% for dementia patients depending on the setting (Khachiyants et al., 2011; Canevelli et al., 2016). However, more recent work suggests a more narrow prevalence between 20 and 27.8% (Angulo Sevilla et al., 2018; Pyun et al., 2019).

Research done during the 1990s and 2000s led some to question whether sundowning represents an actual time-dependent worsening of behavioral disturbances, or instead an increase in caretakers’ perceptions of the stress caused by these disruptions at a particular time of day (Gallagher-Thompson et al., 1992; Bliwise et al., 1993; Cohen-Mansfield, 2007). Additionally, some studies did not find support for an exacerbation of behavioral symptoms occurring specifically around sunset (Bliwise et al., 1993; Friedman et al., 1997), with one suggesting that peak agitation actually occurs during the early afternoon (Martin et al., 2000). However, in a later discussion of diagnostic criteria for sleep disorders in AD (Yesavage et al., 2003), several of these same authors noted that “other research does support the notion that the nocturnal hours or the period of sunset (ranging from 4:00 to 8:00 PM depending on the study) are vulnerable to agitation,” and that “(t)aken together, these results lend support to the existence of a circadian rhythm for agitated behaviors in many AD patients that peaks late in the day, although its precise delineation in real time and its association with sunset, sleep, and patient and/or disease characteristics remain unclear.” Yesavage et al. (2003) further stressed the important point that “although there is mixed evidence for the existence of sundowning and it may be useful descriptively, the term, when used to define sleep disturbance, is too broad to be of practical diagnostic value.” Altogether, this raises the possibility that the sundowning phenomenon reflects a time-dependent disturbance in emotional regulation rather than a direct sleep disturbance. And, its occurrence may be more generally tied to a 4-h window within the late afternoon and early evening instead of being directly tied to sunset.

Indeed, during this same time, numerous more groups reported disturbances in AD and dementia patients that temporally and qualitatively match the traditional description of sundowning-related agitation and aggression (Martino-Saltzman et al., 1991; Cohen-Mansfield et al., 1992; O’Leary et al., 1993; Burgio et al., 1994; Sloane et al., 1998). Even more recently, several observational studies defining sundowning as an increase in neuropsychiatric behaviors (including agitation and aggression) in the late afternoon and early evening have observed this phenomenon in AD patients in association with important circadian or AD-related factors (Silva et al., 2017; Angulo Sevilla et al., 2018; Menegardo et al., 2019; Pyun et al., 2019; Shih et al., 2019). For instance, Menegardo et al. (2019) associated the aggressiveness and irritability of sundowning with increased nocturnal behavior such as wandering. Silva et al. (2017) also associated sundowning with increased depressive and cognitive symptoms, suggesting that multiple emotional systems are disrupted in this syndrome and that these become even more compromised as AD progresses with more associated cognitive decline. Angulo Sevilla et al. (2018) also noted an association of such sundowning symptoms with an increased severity of dementia, but also in association with insomnia and hypersomnia. Interestingly, Pyun et al. (2019) found a strong association between these sundowning symptons and the presence of the apolipoprotein E (APOE) ε4 allele, an important genetic risk factor in the development of late-onset AD that promotes amyloid pathology (Corder et al., 1993).

A recent scoping review across 23 studies focused on sundowning found that temporal periodicity was the most prevalent finding, with 90.0% of the studies that met their criteria for inclusion reporting an onset of behavioral disturbances occurring during the middle afternoon and early night (Boronat et al., 2019). The symptoms examined across these studies most commonly clustered into “psychomotor disturbances” at 83.3%, and included agitation, aggression, and restlessness, followed by a cluster of symptoms categorized as “cognitive disturbances” at 66.7% including confusion, disorientation, and wandering. Importantly, these studies also largely support the notion that sundowning may reflect a time-dependent disturbance in emotional behaviors, rather than a sleep disturbance *per se*. Therefore, in order to better understand and treat sundowning symptoms, it is important to recognize the interacting neural components that modulate the production and daily timing of emotional behavioral states. Interestingly, Todd et al. (2018) found that disrupting the SCN → SPZ → VMH pathway led to increased behavioral aggression specifically during the early resting phase (the light phase for nocturnal mice), a time which appears to be temporally analogous to when AD and dementia patients have traditionally been reported to display sundowning symptoms (Todd et al., 2018). Such shared neural pathways may be promising targets for treatments that could greatly reduce sundowning and other symptoms associated with circadian dysfunction. This review examines the existing literature on specific pathways emanating from the circadian system and the behaviors they regulate, in addition to pathways that provide input to the circadian system and influence its function (Figure 1). It also focuses on the evidence concerning whether AD-related disruption of these circuits might underlie sundowning symptoms, as well as how these pathways might inform potential treatments options.

## The Extended Mammalian Circadian Timing System

The SCN (see Figure 1) is required for daily rhythms of physiology and behavior (Moore and Eichler, 1972; Stephan and Zucker, 1972), and SCN neurons function as individual oscillators with rhythms of electrical activity that have period lengths of about 24 h (Welsh et al., 1995). This electrical activity becomes highly coupled across SCN cells, resulting in an emergent ensemble circadian period (Herzog et al., 1998). The electrical activity rhythms within individual SCN neurons are under the control of canonical “clock genes,” via a transcriptional-translational-post-translational negative feedback loop (Gekakis et al., 1998; Jin et al., 1999). This genetic machinery has been found to be present in cells throughout the brain and body, however, the integrity of the SCN is necessary to synchronize these peripheral oscillators and maintain rhythmic behavior (Mohawk et al., 2012). Specifically, SCN neuronal activity has been shown to be required for such circadian output, as the application of tetrodotoxin to the SCN *in vivo* reversibly disrupts circadian behavior, even while proper circadian timekeeping within the SCN remains intact (Schwartz et al., 1987).

Suprachiasmatic nucleus neurons are predominately GABAergic (Liu and Reppert, 2000), with subpopulations that differentially release several neuropeptides, including vasoactive intestinal peptide (VIP), arginine vasopressin (AVP), gastrin-releasing peptide (GRP), neuromedin S (NMS), and cholecystokinin (CCK). Some of these neuropeptides are arranged somatotopically, as the SCN is composed of “core” and “shell” subregions that express VIP and AVP, respectively (Abrahamson and Moore, 2001). The VIP neurons within the SCN core receive direct retinal input and are required for normal circadian rhythmicity (Harmar et al., 2002; Aton et al., 2005; Maywood et al., 2006). These VIP core neurons then appear to entrain the rhythmicity of AVP shell neurons and other SCN neuronal cell types in order to establish SCN-level synchrony (Aton et al., 2005; Maywood et al., 2006). The subpopulation of SCN neurons expressing NMS have also been implicated as playing a crucial role in circadian pacemaking (Lee et al., 2015), however, more recent work suggests that the critical neurons in this role belong to a molecularly distinct subpopulation that expresses *both* NMS and VIP together (Todd et al., 2020). Interestingly, Todd et al. (2020) found that SCN VIP neurons that also contain NMS are enriched with the transcript *Per2* associated with a core clock gene, whereas the non-NMS subpopulation of SCN VIP neurons that also contain GRP did not have such transcripts. Altogether, this suggests that SCN VIP neurons are composed of both pacemaker and non-pacemaker subpopulations, which is supported by previous work demonstrating that SCN VIP neurons can be divided into two groups based on the light-inducibility of clock genes, innervation of retinal afferents, day-night variability of VIP mRNA, and coexpression of GRP (Kawamoto et al., 2003).

Suprachiasmatic nucleus neurons have been suggested to synchronize downstream molecular clocks and coordinate circadian rhythms via the release of humoral factors, as encapsulated implants of fetal tissue (which prevent the establishment of new neural connections) into SCN-ablated animals have been shown to restore modest behavioral rhythms (Silver et al., 1996). Identified humoral factors that are released by the SCN and have been shown to modulate behavioral and physiological rhythms include transforming growth factor alpha and prokineticin 2 (Cheng et al., 2002; Li et al., 2006; Gilbert and Davis, 2009). However, developmental work suggests that the influence of SCN humoral factors may decrease during the early postnatal period as axonal connections develop between the circadian system and downstream areas regulating behavioral state (Gall et al., 2012; Blumberg et al., 2014). Overall, the SCN’s major axonal output pathway through the SPZ (see below) appears to be the primary method for synchronizing downstream oscillators and maintaining circadian rhythms of behavior (Saper, 2013).

As also depicted in Figure 1, the majority of axons emanating from the SCN synapse onto neurons within the SPZ, an adjacent region of GABAergic cells located just dorsal to the SCN and ventral to the paraventricular hypothalamus (PV) (Watts and Swanson, 1987; Watts et al., 1987; Vujovic et al., 2015). Like the SCN, the SPZ displays circadian rhythms of multiunit activity *in vivo* (Nakamura et al., 2008), and this output pathway has been hypothesized to be the primary circuit by which the SCN synchronizes organismal-level circadian rhythmicity (Saper, 2013). Specifically, studies in rats have shown that circadian rhythms of sleep-wake, locomotor activity, and feeding behavior are regulated by a pathway from the SCN, through the SPZ, to the dorsomedial nucleus of the hypothalamus (DMH) (Lu et al., 2001; Chou et al., 2003). As mentioned previously, it was recently demonstrated that rhythms of aggression propensity in male mice are regulated by SPZ neurons that project to VMH neurons known to promote attack behavior (Todd et al., 2018). These SPZ neurons were found to be active during the early light phase, the resting phase for nocturnal mice, and disrupting their GABAergic transmission resulted in a time-dependent increase in behavioral aggression. Importantly, this time point is temporally analogous to the early resting phase in humans, when sundowning symptoms are most commonly reported in AD patients (Boronat et al., 2019). In addition to aggression, neurons within the VMH have also been associated with the regulation of fear and anxiety (Silva et al., 2013; Kunwar et al., 2015), raising the interesting possibility that the SCN → SPZ → VMH pathway may also influence circadian aspects of these emotional processes in a circadian fashion (Bilu and Kronfeld-Schor, 2013; Albrecht and Stork, 2017). Given the wide range of rhythms that the SPZ appears to influence, this structure appears to be a likely candidate for which its dysfunction, or dysfunction of its inputs (from the SCN or elsewhere), could affect multiple aspects of circadian physiology and behavior that are seen in conditions such as sundowning syndrome.

## Dysfunction Within Major Circadian Structures Associated With Aging and AD Pathology

Interestingly, separate studies have reported conflicting results regarding the direct impact of AD pathology on SCN VIP and AVP neurons in humans. Such studies are often complicated by the fact that researchers often do not have access to both hypothalamic tissue and the profile of circadian behavior of the same patients. However, one study examined hypothalamic tissue containing the SCN in aged patients that had at least 1 week of actigraphy data within 18 months of their death, and found that an age-related decline in VIP, but not AVP, SCN neurons was associated with increased circadian dysfunction (Wang et al., 2015). A group of AD patients examined within this study, however, did not show a significantly greater loss of VIP SCN neurons compared to controls, even though they showed delayed acrophases of the locomotor activity rhythms. This led Wang et al. (2015) to suggest that structures that supply input to the SCN may instead be affected by AD pathology, therefore leading to a disruption of phase-setting and resulting in the delay found in their patients. Indeed, similar phase delays are a common report in AD and dementia patients from several other studies (Harper et al., 2005; Schlosser Covell et al., 2012; Manni et al., 2019). One other study using actigraphy reported that a loss in AVP neurons in the SCN in AD patients was associated with fragmented rhythmicity compared to healthy aged-matched controls (Harper et al., 2008), however, this group used a ratio of AVP neurons to glial cells in only a few selected fields of the SCN, whereas Wang et al. (2015) used a stereological rigorous method to quantify AVP and VIP throughout the entire nucleus. Finally, one other group reported reduced levels of AVP mRNA in the SCN but they did not count AVP mRNA-expressing neurons (Liu et al., 2000), and the same group had previously reported no change in AVP-expressing SCN neurons in elderly dementia patients compared to healthy age-matched controls (Swaab et al., 1985).

Evidence in healthy aging wild-type mice suggests an age-related decline in circadian output from the SCN to the SPZ (Nakamura et al., 2011). These researchers saw a reduction in the circadian amplitude of multi-unit activity rhythms (MUA) in the SCN with age, as well as a similar reduction in the amplitude of MUA rhythms within the SPZ. While the interpretation of these findings as a dysfunction in SCN output is sound, it is possible that these results might also reflect an age-related dysfunction in the SPZ neurons’ ability to maintain rhythms as well (instead of *only* a decline in SCN output). Indeed, the SPZ has been largely overlooked as a possible locus for the circadian dysfunction that has been reported in neurobehavioral pathologies. However, since it regulates both sleep-wake and locomotor rhythms via its projections to the DMH, and also regulates the propensity for aggression via its projections to the VMH, the SPZ is in a logical position to underlie multiple symptoms associated with sundowning should its function become disrupted by AD pathology. It is interesting that, in both rats and mice, the SPZ has been shown to be composed of distinct subregions that differentially project to the DMH or VMH (Vujovic et al., 2015; Todd et al., 2018). Therefore, differences in the degree of dysfunction caused by AD pathology in different SPZ subregions might explain the reported differences in sundowning symptoms across patients, as some studies have reported sundowning to mainly be composed of mood related disturbances such as agitation and aggression, as compared to sleep disturbances, whereas other studies have reported both (Boronat et al., 2019).

## Cholinergic Inputs to the Circadian System

Multiple studies have shown a cholinergic innervation of the SCN (Ichikawa and Hirata, 1986; Kiss and Halasz, 1996; Castillo-Ruiz and Nunez, 2007), and that acetylcholine modulates the function of SCN neurons and circadian rhythmicity (Liu and Gillette, 1996; Liu et al., 1997; Hut and Van der Zee, 2011; Gritton et al., 2013). In the field of AD research, the so-called “cholinergic hypothesis” has long posited that neurodegeneration of neurons in the basal forebrain (BF) expressing acetylcholine, which extensively project to cortical areas, underlie much of the memory and cognitive decline seen during the progression of the disease (Contestabile, 2011; Craig et al., 2011; Pinto et al., 2011). Indeed, there is ample evidence in AD patients that the cholinergic neurons of sub-regions within the BF, such as the nucleus basalis of Meynert (NBM) (Cummings and Benson, 1987; Vogels et al., 1990; Mesulam, 2013; Liu et al., 2015), are a major site of neurodegeneration in AD. Several groups have proposed that a cholinergic BF input to the SCN may also be disrupted in AD, leading to sundowning or other observed circadian deficits (Klaffke and Staedt, 2006; Hut and Van der Zee, 2011; Bedrosian and Nelson, 2013). Lending some support to this hypothesis, acetylcholinesterase inhibitors including donepezil, have been found to ameliorate neuropsychiatric symptoms such as agitation and aggression (Mega et al., 1999; Paleacu et al., 2002; Cummings et al., 2006; Carrasco et al., 2011). One case study also reported that donepezil reduced agitation and restlessness specifically in a sundowning dementia patient (Skjerve and Nygaard, 2000). Interestingly, donepezil has also been shown to enhance rapid eye movement (REM) sleep in AD patients (Moraes Wdos et al., 2006), but has also been associated with an increased prevalence of nightmares (Ridha et al., 2018).

However, the evidence for the existence of a cholinergic pathway from the BF to the SCN comes from only one study in rats using non-specific retrograde tracing from the SCN, and then co-labeling for the cholinergic transporter (CHAT) in BF neurons (Bina et al., 1993). Another study in rats suggested that chemical lesions of the NBM were associated with a reduction in VIP and AVP synthesis and expression in the SCN, however, anatomical tracing was not done in these experiments (Madeira et al., 2004). Importantly, recent work using more selective genetically targeted tracing from the BF in *CHAT-IRES-Cre* mice reported no evidence of such a cholinergic BF to SCN pathway (Agostinelli et al., 2019). While it is possible that this findings represent a species difference between mice and rats, the conservation of a BF to SCN pathway across mammalian species warrants further investigation (Figure 1). Yet, this does not discredit a cholinergic input to the SCN from other areas, such as the brainstem. Indeed, the same authors who reported the cholinergic BF to SCN in rats also reported retrogradedly labeled CHAT cells in the laterodorsal tegmentum (LDT) and pedunculopontine tegmentum (PPT) complex of the brainstem (Bina et al., 1993). The LDT/PPT has been shown to display tau pathology in AD patients, but interestingly, not cholinergic cell loss (Mufson et al., 1988; Dugger et al., 2012; Kotagal et al., 2012). It may be possible that aging and tau pathology could disrupt the function of the LDT/PPT to SCN pathway, even without causing the loss of cholinergic cells, as synaptic changes associated with high levels of soluble of amyloid-β or tau have been reported to appear well before the insoluble plaques or tangles themselves (Scheff et al., 2006; D’Amelio et al., 2011).

In addition to cholinergic neurons, however, the LDT/PPT complex also contains GABAergic and glutamatergic neurons that are known to play different roles in sleep-wake regulation (Kroeger et al., 2017). It is unclear whether these cell populations also project to the SCN and influence circadian function or aggression; a cell type-specific approach to examine the presence of such pathways and their function would be greatly informative. And, although cholinergic cells appear to be spared in the LDT/PPT of AD patients, the presence of tau pathology in this region could instead lead to neurodegeneration of these GABAergic and glutamaterigic populations, which does not appear to have been previously examined. Interestingly, Bedrosian et al. (2011) showed reduced global c-Fos expression (a marker of neuronal activation) in PPT neurons in aged mice compared to healthy adult mice, which was also associated with temporal changes in anxiety behavior. These authors also found similar time-dependent changes in anxiety behavior in APP mice (which bear amyloid-β pathology), but at even earlier ages (however, it does not appear that c-Fos was examined in the PPT in these APP mice).

## Serotonergic Inputs to the Circadian System

There is also substantial evidence for a role in the disruption of serotonergic neurons in AD (Rodriguez et al., 2012; Vakalopoulos, 2017; Chakraborty et al., 2019), and serotonin is also known to play role in circadian regulation (Ciarleglio et al., 2011; Daut and Fonken, 2019). Several studies have suggested a dense serotonergic input from the midbrain raphe complex to the SCN and SPZ (see Figure 1). In hamsters, these serotonergic inputs appear to arise primarily from the median raphe nucleus (MRN) (Meyer-Bernstein and Morin, 1996; Leander et al., 1998; Yamakawa and Antle, 2010), while studies in rats have revealed serotonergic inputs from both the MRN and dorsal raphe nucleus (DRN) (Kawano et al., 1996; Moga and Moore, 1997). Such serotonergic inputs to the SCN appear to play a role in setting circadian phase, as administration of serotonin or serotonergic agonists into the SCN has been shown to produce phase shifts during certain parts of the light-dark cycle (Lovenberg et al., 1993; Ehlen et al., 2001; Sprouse et al., 2004). Additionally, developmental disruption of the serotonin transcription factor *Pet-1* disrupts locomotor activity rhythms and *in vitro* SCN activity (Ciarleglio et al., 2014). Serotonergic function is also highly implicated in the direct regulation of aggression (Nautiyal et al., 2015; Niederkofler et al., 2016), and serotonergic neurons have been shown to project from the raphe complex to the VMH (Kanno et al., 2008). So, its possible that AD-related disruptions in serotonergic signaling also underlie overall levels of aggression, as well as differences at certain times of the day. Indeed, several groups have reported serotonergic deficiencies in AD that were associated with either increased circadian dysfunction or behavioral aggression (Lai et al., 2003; Vermeiren et al., 2014; Chakraborty et al., 2019).

Serotonergic drugs have commonly been administered to AD patients in order to treat aggression, anxiety, and other emotional behavioral disturbances, as well as to treat sleep-wake and circadian disruption. Citalopram, a selective serotonin reuptake inhibitor (SSRI) widely used as an antidepressant, has been found to reduce irritability, anxiety, and aggression in moderately agitated AD patients (Leonpacher et al., 2016; Schneider et al., 2016), however, it was much less effective in the severely agitated patients. Interestingly, patients categorized as the most severely agitated actually showed an increase in nighttime behavioral or sleep disruptions when treated with citalopram (Leonpacher et al., 2016). An intriguing body of work also suggests that early administration of SSRIs may also slow the progression of mild cognitive impairment to AD, perhaps through a mechanism by which serotonin affects the amyloid-β precursor protein, thereby reducing the accumulation of amyloid-β (Elsworthy and Aldred, 2019). It is also possible that serotonin’s modulation of the circadian system could indirectly play a role in slowing or expediting this progression, as chronic circadian dysfunction exacerbates AD pathology (Musiek, 2015). Indeed, trazodone, a serotonin antagonist and reuptake inhibitor (SARI) that is also commonly used as an antidepressant, has been shown to improve circadian function and sleep-wake rhythms in AD patients (Camargos et al., 2014; Grippe et al., 2015). Risperidone and olanzapine, both atypical antipsychotics and antagonists for serotonin (as well as for dopamine), have been shown to have differential effects in AD patients. Risperidone, but not olanzapine, was found to reduce aggression and other neuropsychiatric symptoms in AD patients (Nagata et al., 2017), whereas a separate study in AD patients found olanzapine to reduce anxiety (Mintzer et al., 2001). While serotonin has historically been implicated in sleep-wake regulation, a recent study demonstrated that DRN serotonergic neurons actually promote sleep through anxiolysis, further highlighting the critical role of serotonin in mood and emotional regulation (Venner et al., 2020).

## The Retinohypothalamic Tract

Perhaps the most extensively studied input to the SCN comes from a distinct set of retinal ganglion cells (RCGs) (see Figure 1), via the retinohypothalamic tract (RHT) (Moore et al., 1995). This pathway is required for circadian photoentrainment, as shown by enucleation studies where removing both eyes results in free-running rhythms under a light-dark cycle (Nelson and Zucker, 1981; Foster et al., 1991). A subset set of RGCs are intrinsically photosensitive and contain the photopigment melanopsin (Berson et al., 2002; Hattar et al., 2002; Sekaran et al., 2003). These melanopsin cells themselves comprise 5 different subtypes (M1–M5-type) (Ecker et al., 2010), and evidence suggests that a molecularly distinct subpopulation of M1-type RGCs, defined by their lack of expression of the transcription factor Brn3b and numbering around only 200 cells, are sufficient for driving entrainment of the SCN (Chen et al., 2011). To enable such photoentrainment, the RHT releases glutamate and pituitary adenylate cyclase activating polypeptide (PACAP) onto the SCN (Hannibal et al., 2000). While light during the day is required for proper circadian photoentrainment, light exposure at night has been shown to be deleterious to mood regulation and overall circadian function (Fonken and Nelson, 2014; Bedrosian and Nelson, 2017). Evidence in AD patients suggests altered function of melanopsin RGCs in preclinical AD (Oh et al., 2019), and post mortem studies suggest an AD-related loss of melanopsin RGCs (La Morgia et al., 2016). Bright light therapy has already been shown to improve circadian rhythmicity and mood in AD patients (Figueiro et al., 2014; Munch et al., 2017; Wahnschaffe et al., 2017). Additionally, one study reported that morning light exposure shifted the peak of agitated behavior in patients with severe AD (Ancoli-Israel et al., 2003).

Another interesting possibility for a future treatment of sundowning via this pathway could be using intravitrial injections of chemogenetic vectors into the eye and driving activity of RGCs via peripheral injection of the chemogenetic ligand. A similar strategy has been suggested to show promise as a potential therapy for other mood-related disorders (Bowrey et al., 2017; Venner et al., 2019). Interestingly, the RHT has been shown to densely project to the SPZ in some species, but not in others (Figure 1), and this pathway has been suggested to play a role in modifying nocturnal versus diurnal sleep-wake behavior in a species typical manner (Todd et al., 2012). Understanding how such species differences impact circadian function will be vital for teasing apart the underlying factors contributing to circadian phase preference (diurnality versus nocturnality), which will be critical for properly translating the findings of AD-related research in nocturnal rodents into potential treatment applications in diurnal AD patients.

## The Geniculohypothalamic Tract

As also depicted in Figure 1, the SCN and SPZ are also known to receive input from photo receipient structures in the thalamus, the ventral lateral geniculate nucleus (LGN) and the adjacent intergeniculate leaflet (IGL), via the geniculohypothalamic tract (GHT) (Moore et al., 2000). Importantly, AD patients have been reported to show significant amyloid-β pathology in the LGN (Erskine et al., 2016). The GHT pathway releases GABA and neuropeptide Y (NPY), and this input has been shown to influence the response of SCN neurons to light, as well as to play a critical role in non-photic entrainment (Mrosovsky, 1996; Harrington, 1997). For instance, giving rodents time-dependent access to a novel running wheel leads to non-photic phase advances and ultimately entrainment of the circadian system (Reebs and Mrosovsky, 1989). NPY released from the IGL neurons that make up the GHT appears to underlie these non-photic effects on the SCN, as novelty-induced wheel running induces c-Fos expression in the IGL NPY neurons (Janik and Mrosovsky, 1992), and infusions of NPY directly into the SCN produce similar phase shifts (Albers and Ferris, 1984; Huhman and Albers, 1994). To further support this view, electrolytic lesions of the IGL (Janik and Mrosovsky, 1994), and SCN infusion of NPY antiserum (Biello et al., 1994), both block the phase-advancing effect produced by novelty-induce wheel running. Interestingly, these results may suggest a pathway by which daily exercise at a consistent time could improve circadian function and reduce possible sundowning symptoms in AD and dementia patients, which has previously been suggested as a strategy to counteract attenuation of circadian rhythms that come with normal aging and AD (Duncan, 2020). Lending support to this idea, timed access to a running wheel has already been shown to increase the robustness of circadian behavioral rhythms in mice lacking the VIP receptor, VPAC2 (Power et al., 2010). Additionally, two separate studies have indicated that daily exercise enhances circadian cortisol rhythms in patients with AD or mild cognitive impairment (Tortosa-Martinez et al., 2015; Venturelli et al., 2016). Moreover, one group found that routine walking at certain times of the day ameliorated sundowning symptoms in AD patients in two separate studies (Shih et al., 2017; Shih et al., 2019).

## Other Neurotransmitter Inputs to the SCN: Dopamine and Orexin

While less is known about their potential role in AD and related dementias, there is some evidence to suggest that dopaminergic and orexinergic inputs to the SCN may could also be comprised in these neurodegenerative diseases. For instance, recent work has implicated an important role for dopaminergic input to the SCN from the ventral tegmental area (VTA) (Grippo et al., 2017). Grippo et al. (2017) demonstrated that this dopaminergic input to the SCN is important for resynchronizing locomotor activity rhythms to shifts of the light-dark cycle, and that elevating levels of dopamine in the SCN actually accelerates photoentrainment. Interestingly, work in a transgenic mouse model of AD pathology revealed degeneration of VTA dopaminergic neurons (Nobili et al., 2017), and a imaging study in prodromal AD patients suggest a decrease in VTA volume (De Marco and Venneri, 2018).

Additionally, the neurotransmitter orexin, located in the lateral hypothalamus and perifornical region (de Lecea et al., 1998), is known for its role in maintaining consolidated wakefulness as the degeneration of orexinergic neurons results in the sleep disorder narcolepsy (Lin et al., 1999). Orexinergic fibers have also been shown to project to the SCN (Backberg et al., 2002), and have been shown to modulate SCN activity (Belle et al., 2014). Belle et al. (2014) found that orexin is upregulated at dusk in nocturnal mice, and suppresses the activity of SCN neurons that specifically express the clock gene *Per1*. These authors also demonstrated that orexin enhances the resetting ability of NPY in the SCN (that has been released from the IGL), highlighting how multiple input pathways may act together to modulate circadian rhythmicity. Interestingly, AD patients with high levels of neuropsychiatric symptoms, including agitation and aggression, have been found to have higher overall levels of orexinergic tone and fragmented sleep (Liguori et al., 2018). Indeed, other studies have found that similarly high cerebrospinal fluid (CSF) levels of orexin in AD patients are also associated with increased amyloid-β levels (Gabelle et al., 2017). While promising, more work is needed to better understand the role of orexinergic and dopaminergic influence on the circadian system in AD, in order to delineate their respective potential contributions to sundowning symptoms.

## Summary

Several characteristic non-cognitive symptoms of AD and related dementias involve behavioral and physiological processes known to be regulated by the hypothalamus (Ishii and Iadecola, 2015; Hiller and Ishii, 2018). These include, among others, circadian and sleep-wake dysfunction, and emotional behavioral disruptions such as agitation and aggression. These particular non-cognitive symptoms are comorbid in the clinical phenomenon known as sundowning syndrome. Whether this term is an appropriate descriptor is debatable, given that the direct linkage of this phenomenon to sunset is not always supported (Yesavage et al., 2003). However, the weight of the evidence does suggest that a time-dependent exacerbation of emotional behavioral disturbances, including agitation and aggression, is prevalent in AD and dementia patients during the late afternoon and early evening (Boronat et al., 2019). Interestingly, this phenomenon seems to be less connected to sleep disruption, *per se*, and more directly tied to disturbances in emotional state.

Although sundowning has been studied for several decades, its cause remains unclear. Evidence from basic research (Bedrosian and Nelson, 2013; Todd et al., 2018), along with pathological findings from AD patients (Wang et al., 2015; Erskine et al., 2016; Chakraborty et al., 2019), suggests several pathways that might be involved in the circadian dysfunction, and agitation and aggression, underlying sundowning. There is a strong association between circadian rhythms and emotional regulation (Hood and Amir, 2018; Ketchesin et al., 2020), and the shared circuitry between these two systems presents potential candidates for such pathology-related dysfunction. These include the major circadian structures themselves, the SCN, SPZ and its output pathways to the DMH and VMH (Venner et al., 2019), as well as several structures that project to circadian system.

Disrupting GABAergic transmission from SPZ cells that project to the VMH has been shown to cause increased behavioral aggression during the early resting phase, when these cells have been shown to be active in a time dependent manner (Todd et al., 2018). There is also some evidence for LDT/PPT dysfunction associated with a time-dependent change in anxiety in aged mice (Bedrosian et al., 2011) (but it is unclear whether the affected cells are cholinergic, glutamatergic, or GABAergic), and LDT/PPT neurons also to project to the SCN (Bina et al., 1993). Serotonergic function seems to be dysregulated in AD (Rodriguez et al., 2012; Vakalopoulos, 2017), and serotonergic neurons of the midbrain raphe complex project to and modulate the circadian system and are highly involved in mood regulation and aggression (Ciarleglio et al., 2011; Niederkofler et al., 2016; Daut and Fonken, 2019). Direct retinal input to the SCN, as well as the direct NPY input from the retinorecipient IGL, are also possible candidates as pathology and dysfunction have been reported in these structures in AD (Erskine et al., 2016; La Morgia et al., 2016), and properly timed light exposure improves circadian rhythms and mood whereas ill-timed light exposure has deleterious effects (Bedrosian and Nelson, 2017). Altogether, a better understanding of the role of these pathways in behavioral and emotional timing will be important for treating circadian dysfunction and sundowning-related symptoms in AD and dementia patients and may also lead to the identification of important early indicators of the progression of AD. For instance, agitation and aggression have been found to be important predictors of the progression from mild cognitive impairment to probable AD, suggesting such behavior could be an important early indicator that could inform treatment options (Dietlin et al., 2019). Similarly, circadian dysfunction of locomotor activity has been shown to be present in preclinical AD patients, well before the cognitive and amnesiac symptoms appear (Musiek et al., 2018). Thus, such interventions hold the promise of improving quality of life for both patient and caregiver, and may even slow the progression of the disease.

## Author Contributions

The author confirms being the sole contributor of this work and has approved it for publication.

## Conflict of Interest

The author declares that the research was conducted in the absence of any commercial or financial relationships that could be construed as a potential conflict of interest.
